# Bayesian Inference in Auditing with Partial Prior Information Using Maximum Entropy Priors

**DOI:** 10.3390/e20120919

**Published:** 2018-12-01

**Authors:** María Martel-Escobar, Francisco-José Vázquez-Polo, Agustín Hernández-Bastida

**Affiliations:** 1 Department of Quantitative Methods, University of Las Palmas de Gran Canaria, 35001 Las Palmas de Gran Canaria, Spain; 2 Department of Quantitative Methods, University of Granada, 18071 Granada, Spain

**Keywords:** auditing, Bayesian inference, dollar unit sampling, modified likelihood, partial prior information

## Abstract

Problems in statistical auditing are usually one–sided. In fact, the main interest for auditors is to determine the quantiles of the total amount of error, and then to compare these quantiles with a given *materiality* fixed by the auditor, so that the accounting statement can be accepted or rejected. Dollar unit sampling (DUS) is a useful procedure to collect sample information, whereby items are chosen with a probability proportional to book amounts and in which the relevant error amount distribution is the distribution of the taints weighted by the book value. The likelihood induced by DUS refers to a 201–variate parameter p but the prior information is in a subparameter θ linear function of p, representing the total amount of error. This means that partial prior information must be processed. In this paper, two main proposals are made: (1) to modify the likelihood, to make it compatible with prior information and thus obtain a Bayesian analysis for hypotheses to be tested; (2) to use a maximum entropy prior to incorporate limited auditor information. To achieve these goals, we obtain a modified likelihood function inspired by the induced likelihood described by Zehna (1966) and then adapt the Bayes’ theorem to this likelihood in order to derive a posterior distribution for θ. This approach shows that the DUS methodology can be justified as a natural method of processing partial prior information in auditing and that a Bayesian analysis can be performed even when prior information is only available for a subparameter of the model. Finally, some numerical examples are presented.

## 1. Introduction

This paper addresses the statistical problem of estimating the total amount of error in an account balance obtained from auditing. To do so, the statistical toolbox employed by the auditor must be adapted to use a Bayesian approach. The conclusions drawn from the audit process are commonly based on statistical methods such as hypothesis testing, which in turn is based on compliance testing and substantive testing. The first of these is conducted to provide reasonable assurance that internal control mechanisms are present and function adequately. Substantive testing seeks to determine whether errors are present and if so, their size. In auditing practice, the total amount of error in a single statement, denoted by θ, and associated substantive testing are highly important to decision making. For instance, the test $H_{0}:\theta \leq \theta_{m}$ vs. $H_{1}:\theta >\theta_{m}$, can be conducted in order to accept or reject the amount of error detected in the audit, where $\theta_{m}$ denotes the total amount of error the auditor deems material. Johnstone (1995) [1] presented auditing evidence showing that the classical hypothesis test is incoherent and that Bayesian techniques are to be preferred.

Monetary Unit Sampling (MUS) or equivalently Dollar Unit Sampling (DUS, is commonly used to obtain sample information. In DUS, the population size is the recorded book value (*B*) and the sample plan consists of selecting monetary (dollar) units with an equal chance of being selected. The amount of error for each dollar selected is the difference between its book value and its audit value. The taint of the randomly–selected dollar unit is given by the quotient between the error and the book values. Most of the audited values will be correct and so the associated errors will be zero. The taints in a dollar unit sample are recorded and used to draw inferences about the parameter of interest, i.e., the total amount of error. In practice, auditors usually assume that no amount can be over or under–estimated by an amount greater than its book value. Therefore, the range of taints extends from −100 to +100 per cent in increments of one per cent: −100,−99,…,−1,0,1,…,99,100, and the proportions of each taint are: $p_{-100},p_{-99},\ldots ,p_{-1},p_{0},p_{1},\ldots ,p_{99},p_{100}$. For a sample DUS of size n, the practitioner knows the observed number of tainted dollar units in the sample with *i*% taints, $n_{i}$, where $0\leq n_{i}\leq n,$i=−100,…,0,…,100, and $\sum_{i=-100}^{100}n_{i}=n$. In practice, *B* is very large in relation to sample size *n* and then the multinomial model adequately reflects the likelihood function. The likelihood of the problem is expressed as a parameter $p=(p_{-100},\ldots ,p_{100})$ of dimension 201,

(1) $$f\left(n|p\right)=\frac{n!}{n_{-100}!\cdot \ldots \cdot n_{100}!}\prod_{i=-100}^{100}p_{i}^{n_{i}},$$

where $n=(n_{-100},\ldots ,n_{100})$.

To complete a Bayesian analysis, a prior distribution is required, and this is frequently a conjugated Dirichlet prior. However, there are certain difficulties. On the one hand, quantifying the expert’s opinion as a probability distribution is a difficult task, especially for complex multivariate problems. Furthermore, although the auditor usually has an intuitive understanding of the magnitude, i.e., the total amount, of error θ, the proportion of $p_{i}$ will be unknown. Finally, the likelihood of the observed data depends on the parameters $p_{-100},\ldots ,p_{100}$. In consequence, the analyst must consider a Bayesian scenario under partial prior information, and seek to combine prior information about θ with the sample information about the individual proportions.

In a non–Bayesian context, McCray (1984) [2] introduced a heuristic procedure to obtain a maximum likelihood function. Following Hernández et al. (1998) [3], we now propose a modification of the likelihood to make it compatible with prior information on θ and then perform a Bayesian analysis. The prior distribution for the total amount of error in the population is commonly asymmetrical and right tailed, and statistically–trained auditors can readily elicit values such as the mean and/or certain quantiles. In this paper, we propose to use for the prior the maximum entropy prior with a specified mean. The advantages of this objective “automatised” prior are that it requires only a small amount of prior information, and nothing else, and is computationally feasible.

The remainder of the paper is organized as follows. Section 2 outlines technical results needed to derived the modified likelihood we use to combine with prior distributions. Section 3 shows how maximum entropy priors can be incorporated into the auditing context. Section 4 then presents some numerical illustrations of the method, and the results obtained are discussed in Section 5.

## 2. The Likelihood Function

Assuming the joint probability mass function given in (1) and consider that there exists a measurable function $\psi (p_{-100},\ldots ,p_{100})=\theta$ such that the auditor has prior information about θ∈Θ and Θ a discrete set of values of θ. Observe that by construction

(2) $$\theta =\psi \left(p_{-100},\ldots ,p_{100}\right)=\frac{B}{100}\sum_{i=-100}^{100}ip_{i}.$$

The following notation will be used. Π denoted a separable metric space, A is the natural σ-field of subsets of Π, and B a sub-σ-field of A, $A_{b}^{+}\left(p\right)$ denotes the set of all real–valued functions f(p),p∈Π, which are nonnegative, bounded and A-measurable, π is a probability measure on (Π,B), $\int_{\Pi }^{*}f\left(p\right)\pi \left(dp\right)$ for $f\in A_{b}^{+}\left(p\right)$, is the upper–integral of f(p) with respect to π [4] and $1_{C}\left(\cdot \right)$ is the indicator function of the set C,

(3) $$1_{C}\left(c\right)=\left\{\begin{array}{cc} 1, & \;\mathrm{if}\;c\in C\\ 0, & \mathrm{otherwise} \end{array}\right.$$

Theorem A1 ([5,6]) in Appendix provides a modified likelihood function for the subparameter θ. The function $f_{\pi }^{B}$ in Theorem A1 is the modified likelihood function desired. In fact, we have a A-measurable likelihood function $\psi (p_{-100},\ldots ,p_{100}),$ with A the usual Borel σ-field and also we have prior information given on Θ with its usual σ-field. As Θ is discrete, all atoms of its σ-field are {θ}, and therefore the sets

$$\psi^{-1}\left(\left\{\theta \right\}\right)=\left\{\left(p_{-100},\ldots ,p_{100}\right)\in \Pi \;:\;\psi \left(p_{-100},\ldots ,p_{100}\right)=\theta \right\}$$

are belonging to the σ-field A. Let B be the sub-σ-field of A induced by ψ on Π. If we define the probability of a set $\psi^{-1}\left(\left\{\theta \right\}\right)$ by the probability of {θ} (known a priori), we will have a probability measure on the sub-σ-field B, denoted by π. Furthermore, the sub-σ-field is generated by a countable partition, and in consequence the modified likelihood is given by

(4) $$f^{B}\left(p\right)=\sum_{\theta }\underset{p\in \psi^{-1}\left(\left\{\theta \right\}\right)}{\sup}f\left(p\right)\cdot 1_{\psi^{-1}\left(\left\{\theta \right\}\right)}\left(p\right),$$

where for simplicity we write f(p) to refer function in (1). Observe that function in (4) is constant on every set $\psi^{-1}\left(\left\{\theta \right\}\right)$, and thus we can write the modified likelihood as

(5) $$f^{B}\left(\theta \right)=\underset{p\in \psi^{-1}\left(\left\{\theta \right\}\right)}{\sup}f\left(p\right).$$

Also we note that expressions (4) and (5) are similar to empirical likelihood functions [7] and with the likelihood *induced* in the notation introduced by Zehna (1966) [8]. Likelihood function in (5) is a B-measurable function and compatible with the prior π, thus Bayes’ theorem now apply as

(6) $$\pi \left(\theta |\mathrm{data}\right)=\frac{f^{B}\left(\theta \right)\cdot \pi \left(\theta \right)}{\int_{\Theta }f^{B}\left(\theta \right)\pi \left(d\theta \right)}.$$

We illustrate how to obtain the modified likelihood in (5) with a simulated example.

**Example** **1.** *Consider a DUS sample of 100 items which no errors have been discovered 90 times, one taint is 10% in error, one more taint is 90%; and eight taints are −10% in error (understatement error). Also, we assume that the monetary units are drawn from a population of accounts totaling $B=\$10^{6}$. To find the likelihood of a value θ we solve the following optimization problem*

$$\max\frac{100!}{90!\cdot 8!}p_{-10}^{8}p_{0}^{90}p_{10}p_{90}$$

*subject to:*

$$p_{-10}+p_{0}+p_{10}+p_{90}=1,$$

*and*

$$10,000\left(-10p_{-10}+0p_{0}+10p_{10}+90p_{90}\right)=\theta$$

*and that all proportions are nonnegative and less than one.*

*For example, for a total amount of error θ = 12,000 the proportions obtained are $p_{-10}=0.075,p_{0}=0.894,p_{10}=0.011$ and $p_{90}=0.020$, and the likelihood of this error is 0.014. All computations are easily obtained with Mathematica^©^ using the command NMaximize.*

## 3. The Maximum Entropy Priors

To apply Bayesian methods in auditing, a prior distribution must be assigned to the total error parameter θ. References [9,10], among others, have described how this might be done. In practice, however, Bayesian methods are not widely used because auditors frequently find it difficult to assess a prior probability function. They often lack statistical expertise in this respect, and so cannot easily assess hyperprior parameters, which might not have an intuitive meaning. In most cases, only certain descriptive summaries, such as the mean and/or median of a probability distribution, can be assigned straightforwardly. Thus, auditors tend to feel comfortable assessing certain values of the prior distribution and disregard the other possible values of the parameter. In such a situation, the maximum entropy procedure might be an appropriate way to obtain the prior distribution required.

Let the parameter space Θ be an interval $\Theta =[\theta_{L},\theta_{U}]$. It is well known that the probability distribution π which maximizes the entropy with respect to the objective uniform prior on $\left[\theta_{L},\theta_{U}\right]$ subject to partial prior information given by

(7) $$E^{\pi }\left(g_{k}\left(\theta \right)\right)=\int_{\Theta }g_{k}\left(\theta \right)\pi \left(\theta \right)\;d\theta =\mu_{k},\;k=1,\ldots ,m,$$

has the form

(8) $$\pi \left(\theta \right)\propto \exp\left\{\sum_{k=1}^{m}\lambda_{k}g_{k}\left(\theta \right)\right\},$$

where $\lambda_{k}$ are constants to be determined from the constraints in (7). Observe that functions $g_{k}$ can adopt several interesting expressions. For example, for $g_{1}\left(\theta \right)=\theta$ and $g_{k}\left(\theta \right)={\left(\theta -\mu_{1}\right)}^{k},\;k=2,\ldots ,m,$ we have that partial prior information consists of specifying *m* central moments in the distribution. Quantiles are also easy to incorporate considering $g_{k}\left(\theta \right)=1_{\left(\theta_{L},\theta_{k}\right)}\left(\theta \right)$.

For practical applications and illustrative purposes we focus on situations where only the mean $\theta_{0}$ is given, i.e., $g_{1}\left(\theta \right)=\theta$, and $\mu_{1}=\theta_{0}$. In such case,

1. If $\theta_{0}=\frac{\theta_{L}+\theta_{U}}{2}$, then $\pi \left(\theta \right)∼U\left(\theta_{L},\theta_{U}\right)$, that is, the uniform distribution on the interval $\left(\theta_{L},\theta_{U}\right)$.
2. If $\theta_{0}\neq \frac{\theta_{L}+\theta_{U}}{2}$, then
  (9) $$\pi \left(\theta \right)\propto \exp\left\{\lambda \theta \right\},\;\theta_{L}\leq \theta \leq \theta_{U},$$
  where λ is obtained by solving the nonlinear equation
  (10) $$\theta_{U}+\frac{\left(\theta_{L}-\theta_{U}\right)\exp\left\{-\lambda \theta_{L}\right\}}{\exp\left\{-\lambda \theta_{L}\right\}-\exp\left\{-\lambda \theta_{U}\right\}}=\theta_{0}$$

## 4. Numerical Illustrations

For illustrative purposes, we present a simulated audit situation in which two auditors have partial prior information about the mean, and are comfortable using a maximum entropy prior in a DUS context. Let us consider the DUS data from an inventory with a reported book value of $B=\$10^{6}$, a sample size of 100 items, and observed taints of 0, 5, 10 and 90 and 94, 4, 1, 1, cases, respectively. In order to decide whether to accept the auditee’s aggregate account balance, the auditors then conduct the statistical hypothesis test of $H_{0}:\theta \leq \theta_{m}$ vs. $H_{1}:\theta >\theta_{m}$, where $\theta_{m}$ denotes an intolerable material error. Assume that a figure of five to seven per cent over the reported book value is a common value for this materiality. For instance, let us suppose that the auditors wish to test

(11) $$H_{0}:\theta \leq \$50,000\;vs.\;H_{1}:\theta >\$50,000,$$

that is, $\theta_{m}$ = $50,000.

Following (5), for every value of the total error θ the modified likelihood associated with the DUS data is obtained solving

$$\max p_{0}^{94}p_{5}^{4}p_{10}p_{90}$$

subject to:

$$p_{0}+p_{5}+p_{10}+p_{90}=1,$$

and

$$10,000\left(0p_{0}+5p_{5}+10p_{10}+90p_{90}\right)=\theta$$

and that all proportions are nonnegative and less than one. For a given maximum entropy prior on θ, we can now derive its posterior distribution using (6). Using Bayes’ theorem, these priors can then be updated to posteriors conditioned on the data that were actually observed. To facilitate reproducibility of the results presented, a simplified code version is available as Supplementary Material to this paper.

To compare scenarios where non prior or only limited partial prior information is available, and so the auditors must base their decisions on the information in the data, we present the following situation.

Auditor #1 adopts a reference non–informative prior for the parameter θ, i.e., uniform on Θ. Observe that for a constant prior the Bayes’ theorem is applicable because the constant is cancelled out in (6) and the posterior distribution is equivalent to the normalised modified likelihood. Figure shows the posterior distribution (in grey) of the total amount of error for the DUS data given above.

**Figure 1:**
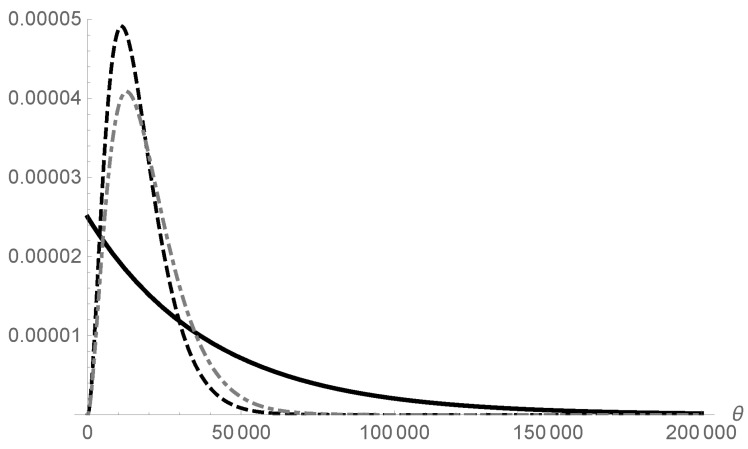
MEP (solid line) and posterior (dashed) distribution of the total amount of error for Auditor #2 in the DUS data example. The posterior distribution associated with the objective uniform prior (Auditor #1) is dotdashed in grey.

On the other hand, the partial prior information provided by Auditor #2 is given by the a priori mean of $\theta_{0}$ = $40,000. With this partial prior information, the maximum entropy prior for θ, deduced by solving Equation (10), corresponds to λ = −25. In practical applications, we suggest using a grid of 1000 total error points for a good approximation to the likelihood function. Figure 1 shows the prior and posterior distribution for Auditor #2 with the sample information considered above. Observe that when just a small amount of prior information is included via the mean, there are differences between the posterior distributions obtained by Auditors #1 and #2. The estimated mean total error, that is, the posterior mean of the distribution in each case, is $16,576.2 and $19,577.2, respectively, and so the posterior distribution for Auditor #1 is more right–skewed than that obtained by Auditor #2.

The posterior probabilities of the null hypothesis are similar, presenting strong evidence for $H_{0}$ [11], although more so under MEP. Table 1 details the posterior probability of the null hypothesis to be tested in (11). All computations were conducted using Mathematica^©^ (version 11.2).

In practice, auditors commonly wish to obtain a high probability quantile of the posterior distribution, say 0.95, and will then accept the accounting balance if this quantile represents a small proportion of the book value, for example no more than five per cent. In Table 1 which shows these quantiles, there is a significant difference between the non-informative and the maximum entropy case, which represent 4.4% and 3.6%, respectively, of the recorded book value. In other words, the posterior probability of the actual total error in the accounting balance being less than $36,000 is 0.95, which represents a reduction of almost 18% in the 95%–quantile compared with a non-informative scenario.

The advantages of the proposed model are highlighted by comparing it with conventional methods such as the conventional Bayesian approach and the classical statistics procedure.

Accordingly, let us first consider a conventional conjugated Bayesian model with multinomial sampling distribution and a non-informative conjugated Dirichlet prior. A burn–in of 10,000 updates followed by a further 50,000 updates produces the parameter estimates $\theta_{0.95}$ = $48,880 and $\mathrm{Pr}\{H_{0}|\mathrm{DUS}\;\mathrm{data}\}=0.954$ (the WinBUGS code is available as Supplementary Material to this paper). Therefore, both of the new Bayesian upper bounds shown in Table 1 are tighter than the above conventional Bayesian bound. Furthermore, the Bayesian Multinomial–Dirichlet model is fairly sensitive to the dimension of p, a concern which does not arise in the proposed formulation. For instance, the above numerical illustration developed with a non-informative Dirichlet prior over the range 0–100 obtains an unrealistic 95% upper bound of $295,900, in contrast with the MEP upper bound which is $36,000.

On the other hand, under a classical approach and following Fienberg et al. (1977) [12], an upper confidence bound for an α percent confidence coefficient with the Stringer method, based on the total overstatement error, is given by

$$B\pi_{0;1-\alpha }+B\sum_{i=1}^{k}\left(\pi_{i;1-\alpha }-\pi_{i-1;1-\alpha }\right)p_{i},$$

where $\pi_{i,1-\alpha }$ denotes the 1−α upper confidence bound for the population proportion when *i* errors are found in the sample. Stringer used a Poisson approximation to obtain these quantities. For the case of this numerical illustration, the bound obtained is $43,950. Therefore, a “classical” auditor can conclude, with at least 95 percent confidence, that the total overstatement error in the population does not exceed $43,950. However, for α = 0.01 we find that the 99 percent upper confidence bound is $62,655, and the null hypothesis in (11) must hten be rejected. This is somewhat confusing, as Johnstone [1] pointed out: “…results close about the *critical* accept/reject partition in conventional hypothesis tests can often result in reversed decisions …”. Furthermore, Table 1 shows that the Bayesian MEP upper bound is tighter than the earlier classical bound.

## 5. Discussion

In this paper, we propose a genuine Bayesian approach as an appropriate formulation for addressing statistical auditing problems. This formulation presents several advantages for the substantive test defined in Section 1: (i) it allows incorporation of the auditor’s judgements about the materiality of the error and makes it easy to derive a reasonable prior distribution; (ii) the Bayesian methodology proposed provides a sensible and straightforward formulation, (iii) the posterior quantities derived are very efficient compared to the existing classical methods, especially when errors are small. The results obtained by our procedure appear to be more reasonable than those achieved by conventional ones such as the classical (Stringer bounds) and the Bayesian (Dirichlet bounds) approaches.

Conventional (conjugated) Bayesian analysis under the DUS methodology, based on multinomial likelihood, needs a Dirichlet prior distribution to be elicited, a requirement which in practice is unrealistic when 201 parameters are involved [13]. Elicitation in a high dimensional parametric space is a complex task [14], but the model presented in this paper overcomes this difficulty. Obviously, alternative priors can be considered. As has been observed elsewhere, objective priors for discrete parameters are starting to be considered both in the univariate scenario [15] and in the multivariate case [16]. This constitutes an interesting line of research for future investigation.

As reported in the NRC Panel review [17], mixture distributions can be appropriate in the audit process. In practice, auditors have found that the distribution of non–zero error amounts differs markedly between types of accounting populations, for instance, between receivables and inventory [18]. This fact introduces additional complexity when we wish to model the audit process without considering the source of information (receivables or not, …). A further advantage of the proposed model is that it includes both over and understatement errors.

The approach we describe in the paper is ”automatic” in the sense that the model incorporates the sample information available and the partial prior information as the prior mean, and no more. No distributional assumptions are required for the likelihood function and no assumptions are made as to the priors. Given this absence of assumptions and the simplicity of the formulation, this approach may be considered reliable for audit purposes. Focusing on hypothesis testing, this paper provides a theoretical basis for using the heuristic quasi–Bayesian model [2]. The numerical illustrations presented suggest that the resulting 95%–quantiles are consistent with the priors and the likelihood considered. In both of these priors (uniform and MEP), the posterior distributions present a moderate right skew towards a higher level of error; only one taint of 90% is observed in the sample and the model is sensitive to this taint. The use of the mean as prior information yields an evident reduction in the 95% upper bound. An interesting area for further investigation of these audit test problems would be to incorporate another intuitive descriptive summary, such as the median or the mode [19].

## Figures and Tables

**Table 1:** Probabilities of the null hypothesis and 95% posterior quantile of the total error in the test problem.

Prior Information	Pr{*H*_0_|DUS Data}	Posterior Quantile *θ*_95_
Non informative	0.97	$44,000
MEP	0.99	$36,000

## Supplementary Materials

The following are available online at http://www.mdpi.com/1099-4300/20/12/919/s1, Mathematica code: Entropy-380586.nb and WinBUGS code: Entropy-380586.odc

## Appendix A

**Theorem** **A1.**
*Let $f\left(p\right)\in A_{b}^{+}\left(p\right)$. Then:*

(a) *There exists a nonnegative real–valued function, finite and B-measurable, denoted by $f_{\pi }^{B}\left(\theta \right)$, defined up to a π-equivalence by*
  $$\int_{F}f_{\pi }^{B}\left(\theta \right)\pi \left(d\theta \right)=\int_{F}^{*}f\left(p\right)\pi \left(dp\right),\;F\in B.$$
(b) *For each $f\left(p\right)\in A_{b}^{+}\left(p\right),$ there exists a version ${\hat{f}}_{\pi }^{B}\left(\theta \right)$ such that:*
  $${\hat{f}}_{\pi }^{B}\left(\theta \right)\geq f\left(p\right)\;\mathrm{and}\;f_{\pi }^{B}\left(\theta \right)\geq f\left(p\right)\;a.s.\;\left[\pi \right]$$
(c) *Some basic properties of the above transformation are:*
  1. *If f(p)≤K, for all p∈Π, then $f_{\pi }^{B}\left(\theta \right)\leq K\;a.s.\;\left[\pi \right]$*
  2. *${\left(\alpha f\left(p\right)\right)}_{\pi }^{B}=\alpha f_{\pi }^{B}\left(\theta \right)\;a.s.\;\left[\pi \right],$ where α is a real constant.*
  3. *Given two functions f(p),g(p), then ${\left(f\left(p\right)+g\left(p\right)\right)}_{\pi }^{B}\leq f_{\pi }^{B}\left(\theta \right)+g_{\pi }^{B}\left(\theta \right)\;a.s.\;\left[\pi \right]$.*
  4. *If the functions f(p),g(p) satisfy f(p)≥g(p), then $f_{\pi }^{B}\left(\theta \right)\geq g_{\pi }^{B}\left(\theta \right)\;a.s.\;\left[\pi \right]$.*
  5. $E_{\pi }\left(f_{\pi }^{B}\left(p\right)\right)=\int^{*}f\left(p\right)\pi \left(dp\right)$.
  6. *If f(p) is a B-measurable function, then $f_{\pi }^{B}\left(\theta \right)=f\left(p\right)\;a.s.\;\left[\pi \right]$.*
(d) *If B is generated by a countable partition $\left\{F_{n}\right\}$, then for each $f\left(p\right)\in A_{b}^{+}\left(p\right)$ the step function defined by*
  $$f^{B}\left(p\right)=\sum_{n}\underset{p\in F_{n}}{\sup}f\left(p\right)\cdot 1_{F_{n}}\left(p\right)$$
  *is a common version of $\left\{f_{\pi }^{B}\left(p\right);\;\pi \in H\right\}$, where H is the class of all probability measures on B.*
